# Development and evaluation of novel tumor-targeting paclitaxel-loaded nano-carriers for ovarian cancer treatment: in vitro and in vivo

**DOI:** 10.1186/s13046-018-0700-z

**Published:** 2018-02-26

**Authors:** Shu Yao, Li Li, Xuan-tao Su, Kai Wang, Zai-jun Lu, Cun-zhong Yuan, Jin-bo Feng, Shi Yan, Bei-hua Kong, Kun Song

**Affiliations:** 1 Department of Obstetrics and Gynecology, Qilu Hospital, Shandong University, 107 Wenhua Xi Road, Jinan, Shandong 250012 People’s Republic of China; 2 Gynecology Oncology Key Laboratory, Qilu Hospital, Shandong University, Jinan, Shandong 250012 China; 3 Department of Biomedical Engineering, School of Control Science and Engineering, Shandong University, Jinan, Shandong 250012 China; 4 Department of Chemistry and Chemical Engineering, Shandong University, Jinan, Shandong 250012 China

**Keywords:** Nano-carriers, Ovarian cancer, Paclitaxel, Tumor targeting, Folic acid

## Abstract

**Background:**

Ovarian cancer is the most leading cause of death and the third most common gynecologic malignancy in women. Traditional chemotherapy has inevitable drawbacks of nonspecific tumor targeting, high toxicity, and poor therapeutic efficiency. In order to overcome such shortcomings, we prepared a novel nano-carrier drug-delivery system to enhance the anti-tumor efficiency.

**Methods:**

In vitro characterizations of nano-carriers were determined by TEM, DLS. Cell viability was measured by MTT method. RT-PCR was performed to measure the expression of FARα in three ovarian cancer cell lines. The drug-release study and the uptaken study were measured in vitro. The pharmacokinetic and the drug distribution study were verified by HPLC methods in vivo. The enhanced anti-tumor efficiency of FA-NP was evaluated by the tumor inhibitory rate in vivo.

**Results:**

Paclitaxel (PTX)-loaded nanoparticles (NPs) (PTX-PEG-PLA-NP and PTX-PEG-PLA-FA-NP) were prepared successfully, and the drug-release study showed that the cumulative release rates of NP groups were much less than free PTX group. The pharmacokinetic study showed that the elimination phase of two kinds of NP groups were much longer than that of PTX group. The drug distribution in different tissues showed that the peak-reach time was 2 h in the PTX group and 6 h in both NP groups. All of these results confirmed the excellent slow-release effects of both kinds of nano-carriers. More importantly, we confirmed that PTX-PEG-PLA-FA-NP had greater uptake by SK-OV-3 cells than PTX-PEG-PLA-NP and free PTX in vitro. A drug-distribution study of tumor-bearing mice demonstrated that the PTX concentration of tumor tissues in the PTX-PEG-PLA-FA-NP group was 3 times higher than the other two groups. PTX-PEG-PLA-FA-NP was uptaken much more by SK-OV-3 cells than PTX-PEG-PLA-NP and free PTX. Eventually, based on the slow-release effect and tumor-targeting characteristics of PTX-PEG-PLA-FA-NP, a cytotoxicity test indicated that PTX-PEG-PLA-FA-NP was much more toxic to SK-OV-3 cells than the controls. The tumor inhibitory rate in the PTX-PEG-PLA-FA-NP group of tumor-bearing mice was about 1.5 times higher than the controls. The tumor targeting and anti-tumor efficiency of PTX-PEG-PLA-FA-NP were confirmed both in vitro and in vivo.

**Conclusions:**

We developed an ovarian cancer targeting nano-carrier drug delivery system successfully, which showed perfect ovarian cancer targeting and anti-tumor effect, thus have the potential to be a new therapy strategy for ovarian cancer patients.

**Electronic supplementary material:**

The online version of this article (10.1186/s13046-018-0700-z) contains supplementary material, which is available to authorized users.

## Background

Ovarian cancer is a leading cause of death and the third most common gynecologic malignancy in women [1, 2]. The 5-year survival rate of advanced stage ovarian cancer patients is presently only approximately 30% [3]. Paclitaxel combined with platinum drugs are the first-line chemotherapy choices for ovarian cancer patients [4]. However, traditional PTX chemotherapy has serious side effects because of the lack of selectively to tumor tissues. To overcome this defect, several strategies such as gene therapy, immunotherapy, and molecular-targeted therapy were developed but have thus shown limited effects in ovarian cancer [5].

In recent years, nanotechnology has shown promising prospects in medicine and made breakthrough progress in the cancer diagnosis and treatment field [6, 7]. Nano-carriers have been widely developed and have shown promising tumor-targeting characteristics [8]. For example, liposomal doxorubicin has achieved good treatment effects in ovarian cancer patients [9, 10]. However, all nano-agents in clinical applications such as liposomal doxorubicin have limited tumor-targeting effects because no active tumor-targeting mechanism exists. Folic acid (FA) plays an important role in cell metabolism and DNA synthesis and repair [11]. Folic acid receptors (FAR) usually over-express in ovarian cancer cells [12, 13]. Therefore, the FA molecule can serve as a specific targeting substance to combine with FAR, which might help us realize the active targeting effect in tumor tissues [14, 15].

In this study, PTX-loaded polyethylene glycol poly (lactic acid) (PEG-PLA) nano-carriers with or without FA molecules (PTX-PEG-PLA-NP and PTX-PEG-PLA-FA-NP) were prepared successfully. The slow-release effects of the nano-carriers were analyzed by drug release experiment in vitro and pharmacokinetic traits as well as tissue-distribution studies in vivo. The uptake experiment in vitro and the tissue-distribution experiment in tumor-bearing animals were performed to demonstrate the tumor-targeting characteristics of PTX-PEG-PLA-FA-NP. Eventually, the anti-tumor efficiency of those two kinds of nano-delivery systems was tested both in vitro and in vivo.

## Methods

### Materials

Loctite, PLA, ethanol, diethyl ether, methanol, tetrahydrofuran (THF), dichloromethane (DCM), polyvinyl alcohol, acetonitrile, epoxy ethane, PTX (99% purity), MTT (3-[4,5-dimethylthiazol-2-yl]-2,5-diphenyltetrazolium bromide), hexamethyl-disiliconate potassium (KHMDS), dimethyl sulfoxide, SYBR Green Master Mix, and FA were purchased from Fule Biological Technology Co., Ltd. (Jinan, China). TRIzol reagent was supplied by Pufei (Shanghai, China). Moloney murine leukemia virus (M-mlV), dNTPs, and RNase inhibitor were obtained from Promega Corp. (Madison, WI, USA).

### Preparation of PEG-PLA-NP and PEG-PLA-FA-NP

In an argon (Ar) gas environment, 10 g of recrystallized lactide was added to conjoined bottle A. THF (20 ml), KHMDS (1.5 ml), and epoxy ethane (2.4 ml) were added to conjoined bottle B, which then was stirred for 2 days. Subsequently, the solution in bottle B was poured into bottle A and stirred for 2 h at 25 °C. Finally, 20 ml of anhydrous acetic acid was added to bottle A and stirred again for 30 min. HCL (0.2 ml) was added to stop the action to form a PEG-PLA block co-polymer. FA was then added for another 24 h of stirring to form PEG-PLA-FA-NP.

### Preparation of PTX-PEG-PLA-NP and PTX-PEG-PLA-FA-NP

PEG-PLA-NP or PEG-PLA-FA-NP (300 mg) dissolved in THF (1 ml) was mixed with PTX (50 mg) dissolved in methylene chloride (1 ml). Subsequently, 3 ml of polyvinyl alcohol was added. The mixed solution was then added to ultrapure water at a speed of 0.4 to 0.6 ml/min and ultra-sonicated at 300 W for 20 min. The emulsion was stirred for 4 h to obtain the precipitate. The PTX-loaded nano-carriers were then ready.

### Characterization of PTX-loaded nano-carriers

The particle size and zeta potential (ZP) of PTX-PEG-PLA-NP and PTX-PEG-PLA-FA-NP were analyzed using 90-Plus Zeta PALS instrument (Malvern Instruments Ltd., Malvern, UK). Transmission electron microscopy (TEM; Tecnai G2F20 S-Twin, FEI Company, Hillsboro, OR, USA) was used to determine the morphology and surface characteristics of the nano-carriers. Nuclear magnetic resonance spectroscopy (HNMR) was used to test the chemical formula of PEG-PLA-NP and PEG-PLA-FA-NP. The amount of PTX encapsulated in the nano-carriers was measured using high-performance liquid chromatography (HPLC, Agilent Technologies, Palo Alto, CA, USA). The drug encapsulation efficacy (EE) and drug loading (DL) were obtained through the following equations:

$$ \mathrm{EE}\ \left(\%\right)=\mathrm{mass}\ \mathrm{of}\ \mathrm{drug}\ \mathrm{incorporated}\ \left(\mathrm{mg}\right)/\mathrm{initial}\ \mathrm{drug}\ \mathrm{added}\ \mathrm{to}\ \mathrm{the}\ \mathrm{medium}\ \left(\mathrm{mg}\right)\times 100\% $$

$$ \mathrm{EE}\ \left(\%\right)=\mathrm{mass}\ \mathrm{of}\ \mathrm{drug}\ \mathrm{incorporated}\ \left(\mathrm{mg}\right)/\mathrm{initial}\ \mathrm{drug}\ \mathrm{added}\ \mathrm{to}\ \mathrm{the}\ \mathrm{medium}\ \left(\mathrm{mg}\right)\times 100\% $$

### Release study of nano-carriers in vitro

The release profiles of PTX from nano-carriers were investigated using the dialysis method. First, PTX-PEG-PLA-NP, PTX-PEG-PLA-FA-NP, and free PTX were dissolved in THF (1 ml) and then placed in a dialysis bag (SnakeSkin, Pierce Biotechnology, Rockford, IL, USA) (3500 KDa) and then immersed in 50 ml of phosphate-buffered saline (PBS). At each predetermined time point (1, 2, 4, 6, 12, 24, 48, 72, 96, 125, and 160 h), 5 ml of PBS was removed and another 5 ml of fresh PBS was added. The PTX concentration in the removed PBS was then analyzed using HPLC to determine the released PTX content.

### FARα expression in three ovarian cancer cell lines

Human ovarian carcinoma cell lines SK-OV-3, HO-8910, and A2780 were supplied by the Gynecology Oncology Key Laboratory of Qilu Hospital. The expression levels of folic acid receptor α (FARα) in 3 ovarian cancer cell lines were determined using real-time polymerase chain reaction (RT-PCR) testing. A2780, SKOV3 and HO8910 cells (STR for A2780, SK-OV-3 and HO-8910 cells were shown in Additional file 1: Figures S1-S3.) were cultured in RPMI 1640 medium with 10% (*v*/v) fetal bovine serum (FBS) and 1% (v/v) penicillin/streptomycin combination. Total RNA was isolated from the above cells using Trizol reagent respectively. Qualitative reverse transcription PCR was performed using Fast-Start Universal SYBR Green Master Mix. Primers for FR gene expression detection were 5′–GAACGCCAAGCACCACAAG–3′ (forward) and 5′–GGTCGACACTGCTCATGCAA–3′ (reverse). Data analysis was performed using the comparative Ct method (2ΔΔCt).

### Cellular uptake study of nano-carriers

Cellular uptake characteristics of two kinds of nano-carriers were analyzed by fluorescence microscopy (Olympus, Tokyo, Japan). Fluorescent marker FITC was combined with PEG-PLA-NP or PEG-PLA-FA-NP. Two kinds of FITC-NP solutions were co-incubated with 3 different ovarian cancer cells for different intervals (1, 2, 4, 6, and 12 h). We normalized the FITC fluorescence to DAPI to test the different cellular densities of different cell types to in the cell uptake experiment. PEG-PLA-FA-NP samples with different FA percentages (5%, 10%, and 20%) were also prepared and co-incubated with SK-OV-3 cells to verify the influence of the FA concentration on cellular uptake. In order to verity FAR-mediated tumor targeting, competition assay using different concentration FA contained culture medium (0 μg/ml, 5 μg/ml, 20 μg/ml and 50 μg/ml in cell culture medium) were performed to test whether PTX-PEG-PLA-FA-NP uptake is FAR-specific on SK-OV-3 cells or not. The intracellular fluorescence images were observed, and the fluorescence intensity was analyzed using Image J software (National Institutes of Health, Rockville, MD, USA).

### Cytotoxicity assay in vitro

The cytotoxicity of the blank NP solution and two kinds of PTX-NPs were assessed by the MTT assay. Different concentrations of the PEG-PLA-NP solutions (0–100 μg/ml) were used to test the cytotoxicity of the blank nano-carriers. Furthermore, standard solutions (PTX, PTX-PEG-PLA-NP, and PTX-PEG-PLA-FA-NP with 10, 25, 50, 75, and 100 μg/ml in PTX concentration) were co-incubated with SK-OV-3 cells at different intervals (12, 24, 36, and 48 h). The MTT test was then performed as described before [16].

### Pharmacokinetic data analysis in rats

Female Sprague-Dawley rats (*n* = 6/group) received intravenous injections of PTX or PTX nano-carriers (6 mg/kg in PTX concentration). Blood samples were collected at predetermined time points (10, 20, 30, and 40 min and 1, 2, 4, 6, 8, 12, 24, 48, and 60 h). We mixed methanol (200 μl) and diethyl ether (2 ml) with plasma (200 μl) samples, vortexed for 5 min and centrifuged at 10,000 rpm for 10 min, then dried the samples under nitrogen conditions. The PTX concentrations were then analyzed using HPLC technology for each sample.

### Tissue-distribution study in tumor-bearing mice

The experimental protocol of the animals in this research was approved by the Animal Ethics Committee of Shandong University (DWLL-2015-001). Healthy female athymic mice (BALB/c nu-nu) (4–6 weeks old) were supplied by the Experimental Animal Center of Shandong University in Jinan, China. SK-OV-3 ovarian cancer cells (1.0 × 10^7^) were implanted subcutaneously or intraperitoneally into the mice to establish subcutaneous or abdominal tumor-bearing models. To establish the standard curve of PTX, blood and tissue samples (liver, spleen, kidney, heart, lung, uterus, small intestine, and tumor tissues) from tumor-bearing mice were homogenized with 5 ml of PBS. Subsequently, 20 μl different quantitative standard PTX solutions (0.95, 1.9, 9.5, 19, 38, 76, and 152 μg/ml) were added to homogenate samples (200 μl), followed by an HPLC test. The linear regression equation was then established based on the PTX concentration (X) and AUC (Y) value from the HPLC test. Abdominal tumor-bearing mice were randomly divided into 3 groups (30 mice/group). Each mouse was intravenously injected with either PTX or PTX nano-carriers solution (6 mg/kg). At predetermined time points (1, 2, 4, 6, 12, and 24 h), 5 mice in each group were euthanized; their plasma and tissue samples were quickly collected for an HPLC test and then calculated based on the linear regression equation mentioned above to determine the PTX concentration in the samples.

### Anti-tumor effect study in vivo

The subcutaneous tumor-bearing animal mice were randomly divided into 4 groups (7/group). A single dose of PTX (6 mg/kg) in free PTX, PTX-PEG-PLA-NP, or PTX-PEG-PLA-FA-NP groups was intravenously administered to each mouse and saline solution was used as a control. Each injection was repeated every 3 days for a total of 6 consecutive injections [17]. The tumor sizes and animal body weights were then measured every 3 days. Tumor size was calculated using the formula *V* = (*ab*^2^)/2, where *a* represents the longest diameter and *b* represents the diameter orthogonal to *a* (mm).

### Statistical analysis

Statistical evaluations of data were performed using Student’s unpaired *t*-test. Statistical analysis was performed using SPSS 20.0 software (SPSS, Inc., Chicago, IL, USA). A value of *p* < 0.05 was considered statistically significant.

## Results

### Characterization of PTX-PEG-PLA-NP and PTX-PEG-PLA-FA-NP

**Fig. 1:**
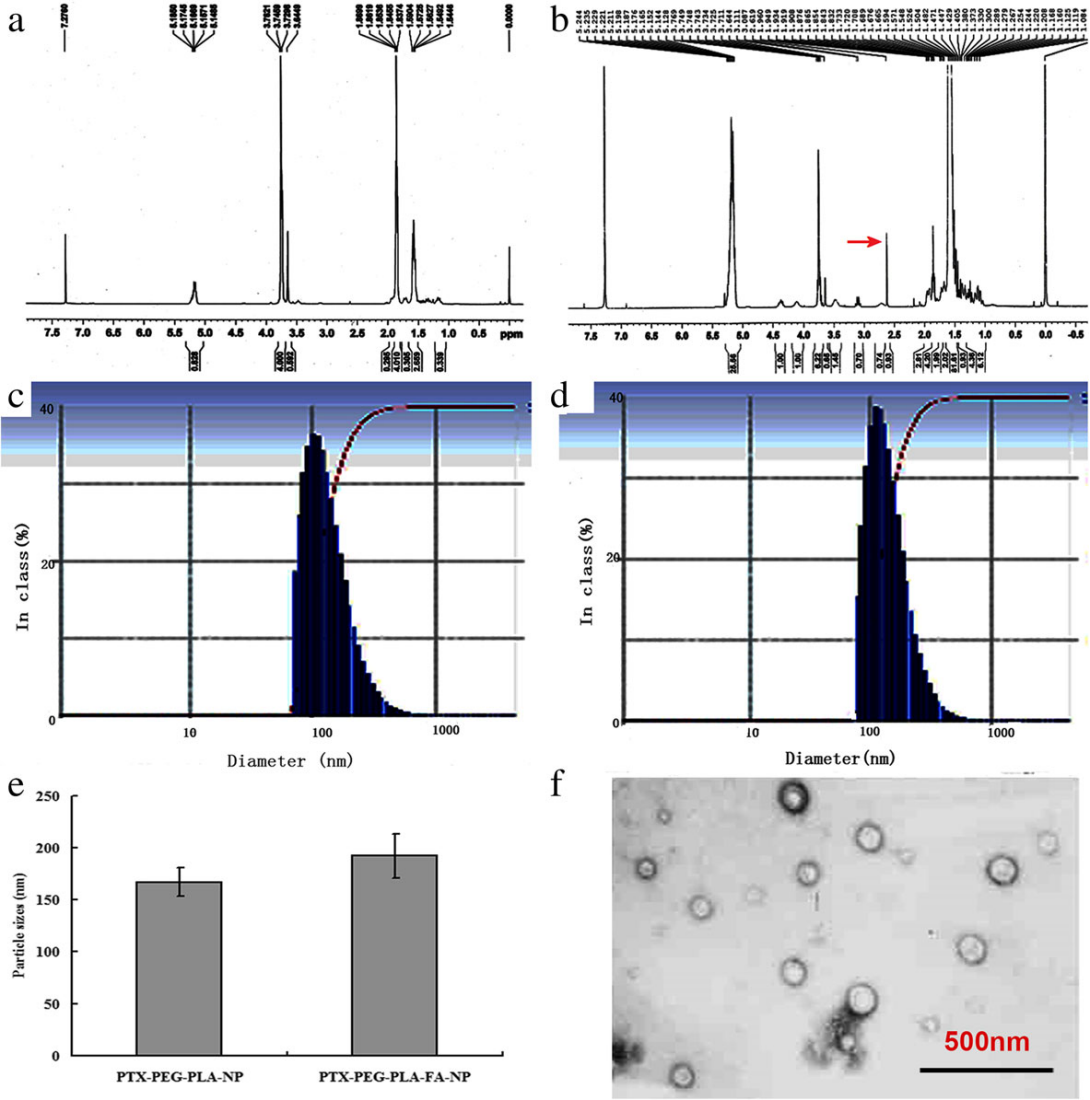
The characterization of PTX-PEG-PLA-NP and PTX-PEG-PLA-FA-NP. Notes: (**a**) HNMR (Nuclear Magnetic Resonance) image of PEG-PLA-NP; (**b**) HNMR image showed that PEG-PLA-FA-NP owned an obvious peak at 2.60 ppm, which indicated that FA was linked to PEG-PLA-NP; (**c**/**d**) Particles size distribution pattern of PTX-PEG-PLA-NP and PTX-PEG-PLA-FA-NP; (**e**) Particles sizes of two kinds of nano-carriers; (**f**) TEM images of PTX-NP, which was well separated, spherical in shape and with smooth surface

Figure 1a/b represents the HNMR feature of PEG-PLA-NP and PEG-PLA-FA-NP. It shows that there was a peak at 2.60 ppm in PEG-PLA-FA-NP compared to PEG-PLA-NP, which successfully confirmed that FA was linked to PEG-PLA-NP. The particle sizes were 167.54 ± 13.80 nm for PTX-PEG-PLA-NP, as shown in Fig. 1c, and 192.32 ± 21.33 nm for PTX-PEG-PLA-FA–NP, as seen in Fig. 1d. Figure 1e shows the particle size of two different nanoparticles, there is no statistically significant of the two types of nanoparticles (*P* > 0.05). Figure 1f shows the TEM images of the nano-carriers, which were well separated, spherical in shape, and had a smooth surface. The ZP values in two kinds of PTX-NPs were − 39.81 ± 3.45 mV and − 18.47 ± 1.45 mV, respectively. The EE was approximately 73.75% for PTX-PEG-PLA-NP and 81.50% for PTX-PEG-PLA-FA-NP, while the amounts of DL were 12.29% and 5.42%, respectively.

### Drug release profiles of nano-carriers

**Fig. 2:**
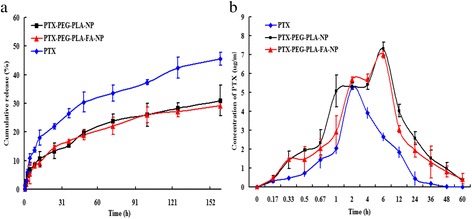
The release profiles and the pharmacokinetics features of different groups. Notes: (**a**) The drug release profiles of free PTX solutions, PTX-PEG-PLA-NP and PTX-PEG-PLA-FA-NP. The PTX cumulative release rate in free PTX group increased markedly over that of other two PTX-NP groups after 4 h, which indicated that the nano-carriers exhibited delayed drug release; (**b**) The pharmacokinetics results in different drug groups. The elimination phase (*t*_1/2,β_) of PTX-PEG-PLA-NP and PTX-PEG-PLA-FA-NP solutions (13.47 h and 15.92 h) were much longer than that of PTX solution (6.41 h, *p* < 0.05). The AUC was 159.81 μg/ml/h for PTX-PEG-PLA-NP and 132.72 μg/ml/h for PTX-PEG-PLA-FA-NP, which were both approximately three-fold greater than that of free PTX (52.65 μg/ml/h)

As shown in Fig. 2a, the drug release rates of the two nano-carriers were 6.98% and 5.44%, which were much less than that of the free PTX solution (10.84%) at 4 h. Moreover, the cumulative release rate of PTX in the free PTX solution increased markedly after 4 h compared with the two PTX-NP groups, which indicated that nano-carriers had the advantage of drug slow-release effect.

### Pharmacokinetic features of different groups

Standard curves were established for calculating the PTX concentrations in various tissues and plasma. Linear regression analysis provided the linear regression equation between the PTX concentration (X) and AUC (Y) (Table 1). Pharmacokinetic features are shown in Fig. 2b. The elimination phases (*t*_1/2,β_) of PTX-PEG-PLA-FA-NP and PTX-PEG-PLA-NP (15.92 h and 13.47 h, respectively) were much longer than those of the free PTX (6.41 h, *p* < 0.05). The AUC was 159.81 μg/ml/h for PTX-PEG-PLA-NP and 132.72 μg/ml/h for PTX-PEG-PLA-FA-NP (*p* > 0.05), which were both approximately 3-fold greater than the free PTX (52.65 μg/ml/h, *p* < 0.05). Based on these results, the PTX loaded into the nano-carriers was cleared much more slowly than the free PTX in blood circulation. The slow-release effect of the nano-carriers was proved again in addition to the results of the drug release study.

**Table 1:** Linear equation of PTX in plasma and other tissues

Tissues	Linear equation	R^2^
Liver	Y = 220.198X + 273.685	0.991
Kidney	Y = 314.886X + 243.909	0.998
Plasma	Y = 282.057X + 56.232	0.999
Heart	Y = 241.086X + 395.958	0.995
Spleen	Y = 312.948X + 288.311	0.994
Lung	Y = 220.198X + 273.685	0.997
Intestine	Y = 267.209X + 460.006	0.998
Ovary	Y = 312.655X + 279.929	0.996
Tumor	Y = 304.006X + 49.557	0.997

### Expression levels of FARα in ovarian cancer cell lines

**Fig. 3:**
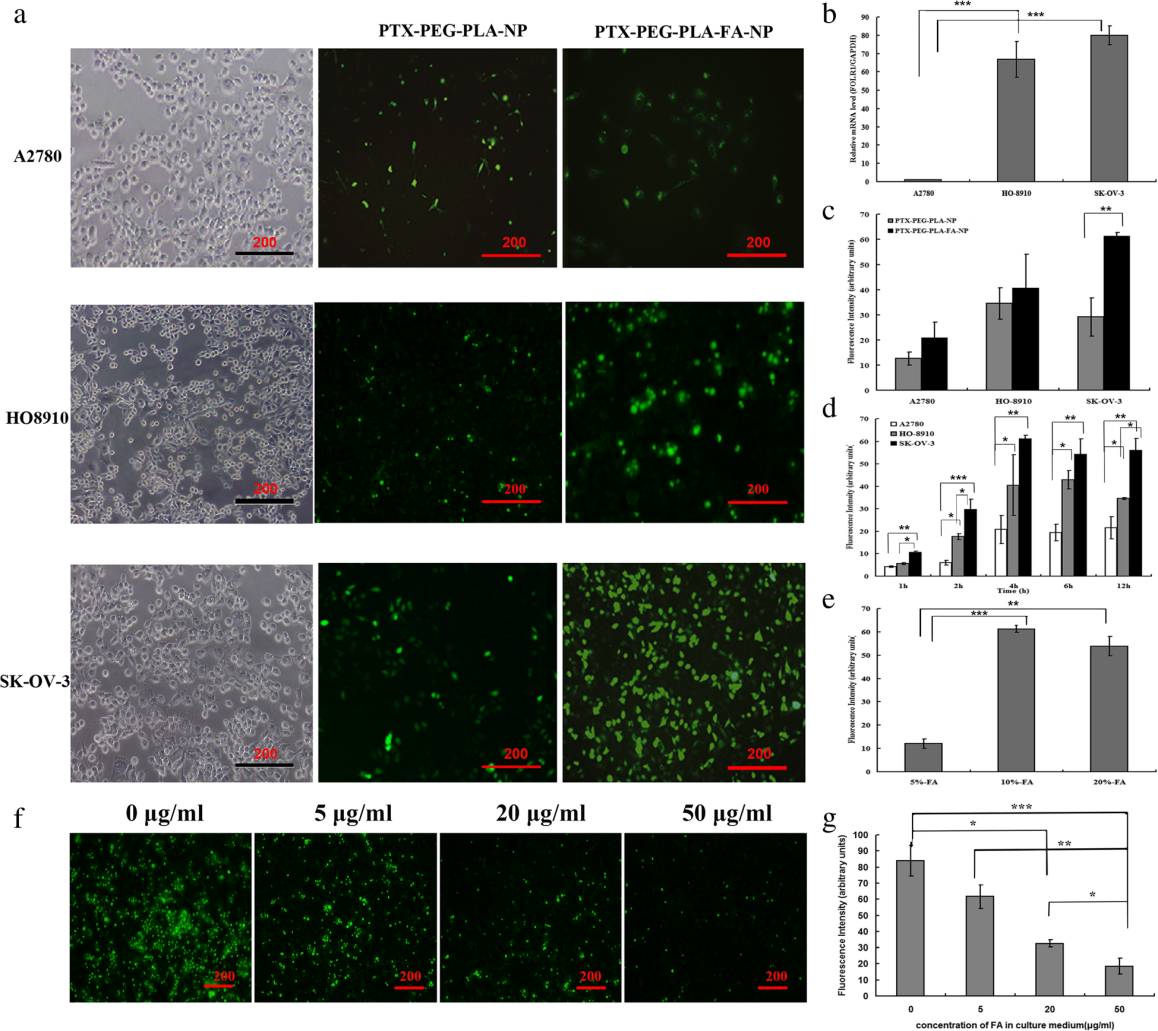
The cellular uptake ability of both PTX-PEG-PLA-NP and PTX-PEG-PLA-FA-NP in SK-OV-3, HO-8910, and A2780 cells under fluorescence microscopy. Notes: (**a**) The cellular uptake amounts of both PTX-PEG-PLA-NP and PTX-PEG-PLA-FA-NP (10% FA) were examined in SK-OV-3, HO-8910, and A2780 cells (co-incubated for 4 h) by fluorescence microscopy. The images showed that more PTX-PEG-PLA-FA-NP was taken up in SK-OV-3 cells than in HO-8910 and A2780 cells. Furthermore, SK-OV-3 cells had taken up more PTX-PEG-PLA-FA-NP than PTX-PEG-PLA-NP; (**b**) The expression levels of FRα in three ovarian cancer cell lines determined by RT-PCR testing; (**c**) Fluorescence density half-qualitatively analysis results in SK-OV-3, HO-8910, and A2780 cells of PTX-PEG-PLA-NP and PTX-PEG-PLA-FA-NP (10% FA contained, incubated for 4 h); (**d**) The fluorescence intensity of PTX-PEG-PLA-FA-NP in three different ovarian cancer cell lines under different incubation time; (**e**) The fluorescence intensity of PTX-PEG-PLA-FA-NP in SK-OV-3 cells with different FA concentration (5%, 10% and 20% FA); Fig. 3f showed the cellular uptake results of PTX-PEG-PLA-FA-NP in SK-OV-3 cells in different concentration FA contained culture medium (0 μg/ml, 5 μg/ml, 20 μg/ml and 50 μg/ml) by fluorescence microscopy; The images showed that more PTX-PEG-PLA-FA-NP was taken up by SK-OV-3 cells in the FA-free culture medium. With FA concentration increase from 5 μg/ml to 50 μg/ml the fluorescence signals in the SK-OV-3 cells decreased accordingly, with suggested that PTX-PEG-PLA-FA-NP uptake were FAR-specific on SK-OV-3 cells; Fig. 3g showed the fluorescence intensity of PTX-PEG-PLA-FA-NP in SK-OV-3 cells with different concentration FA contained culture medium (0 μg/ml, 5 μg/ml, 20 μg/ml, 50 μg/ml). *p < 0.05, **p < 0.01, ***p < 0.001

As shown in Fig. 3b, the FARα levels in the SK-OV-3 cells (79.91 ± 9.78) were much higher than in the other two cells HO-8910 (66.71 ± 5.23) and A2710 (1.00 ± 0.05) (*p* < 0.05). Based on this result, SK-OV-3 was used in the following experiments.

### Cellular uptake results in vitro

As shown in Additional file 2: Figure S1, we normalized the FITC fluorescence to DAPI to test the different cellular densities of different cell types in the cell uptake experiment. The results showed that there were no statistical difference among groups (*p* > 0.05), which indicated that the number of cells in each group was the same. As shown in Fig. 3a, the SK-OV-3 cells took up more PEG-PLA-FA-NP than PEG-PLA-NP. In addition, more PEG-PLA-FA-NP was taken up by the SK-OV-3 cells than the HO-8910 and A2780 cells. The fluorescence intensity of PEG-PLA-NP in three ovarian cancer cell lines (A2780, HO-8910, and SK-OV-3) were 12.62 ± 2.63 a.u., 34.58 ± 6.22 a.u., and 29.19 ± 7.63 a.u. (*p* > 0.05), respectively. In the PEG-PLA-FA-NP group, the fluorescence intensities were 20.83 ± 6.27 a.u., 40.56 ± 13.44 a.u., and 61.245 ± 1.47 a.u. (*p* < 0.05), respectively. In the SK-OV-3 cells, the fluorescence intensity of PEG-PLA-FA-NP was much higher than PEG-PLA-NP, as shown in Fig. 3c, while in the other two cancer cell lines, there were no statistical differences between the two groups (*p* > 0.05). Figure 3d shows that the fluorescence intensity peaked at 4 h in all three kinds of cancer cells. Figure 3e shows that in the SK-OV-3 cells, the PEG-PLA-FA-NP solution with 10% FA had greater uptake than both 5% FA and 20% FA. Based on these results, PEG-PLA-FA-NP-based tumor-targeting characteristics were confirmed in vitro, and several parameters such as the SK-OV-3 cells, 4 h after drug delivery, and 10% FA contents were used in the following experiments. Figure 3f shows the cellular uptake results of PTX-PEG-PLA-FA-NP in SK-OV-3 cells in different concentration FA contained culture medium (0 μg/ml, 5 μg/ml, 20 μg/ml and 50 μg/ml) by fluorescence microscopy; The images showed that more PTX-PEG-PLA-FA-NP was taken up by SK-OV-3 cells in the FA-free culture medium. With FA concentration increase from 5 μg/ml to 50 μg/ml the fluorescence signals in the SK-OV-3 cells decreased accordingly, with suggested that PTX-PEG-PLA-FA-NP uptake were FAR-specific on SK-OV-3 cells; Fig. 3g shows the fluorescence intensity of PTX-PEG-PLA-FA-NP in SK-OV-3 cells with different concentration FA contained culture medium (0 μg/ml, 5 μg/ml, 20 μg/ml, 50 μg/ml).

### Cellular cytotoxicity of nano-carriers in vitro

**Fig. 4:**
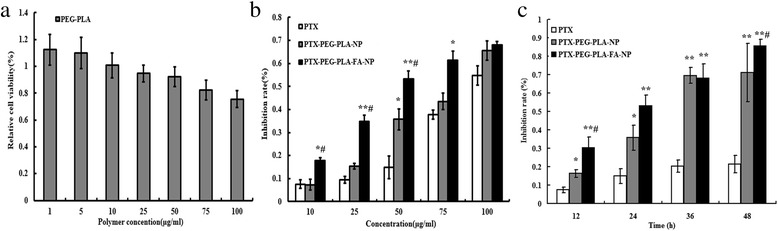
The cellular toxicity of two kinds of nano-carriers in vitro . Notes: (**a**) The toxicity of the blank PEG-PLA-NP to SK-OV-3 cells. Up to the concentration of 100 μg/ml, blank nano-carriers did not inhibit the SK-OV-3 cells viability significantly; (**b**) The cellular inhibition rate in different groups. PTX-PEG-PLA-FA-NP showed significantly more toxicity against SK-OV-3 cells at multiple dosages than PTX-PEG-PLA-NP as well as free PTX based on the equivalent PTX concentrations (*p* < 0.05). (**c**)The toxicity to SK-OV-3 cells under different co-incubation intervals. *p < 0.05, **p < 0.01, ***p < 0.001 vs PTX. ^**#**^*p* < 0.05, ^**# #**^*p* < 0.01, ^**# # #**^*p* < 0.001 vs PTX-PEG-PLA-NP

Figure 4a shows that up to a concentration of 100 μg/ml, PEG-PLA-NP did not obviously inhibit the SK-OV-3 cells, with a relative cell viability rate of 80%, which confirmed the hypotoxicity of PEG-PLA-NP. Figure 4b indicates that PTX-PEG-PLA-FA-NP had more cytotoxicity against the SK-OV-3 cells at multiple dosages than the other two groups given the same PTX concentrations (co-incubated for 24 h) (*p* < 0.05). However, PTX-PEG-PLA-NP showed an equivalent inhibitory efficacy to free PTX (*p* > 0.05). For example, at a PTX dose of 50 μg/ml, the cell inhibition rates were 0.15 ± 0.05, 0.36 ± 0.05, and 0.53 ± 0.04 in the PTX, PTX-PEG-PLA-NP, and PTX-PEG-PLA-FA-NP groups, respectively (*p* < 0.05). Once loaded into PEG-PLA-FA-NP, only a half dose of PTX could achieve the same toxicity as the full dose of free PTX. Figure 4c shows that the toxicities of the three different groups (PTX concentration was 50 μg/ml) were time-dependent and the cytotoxicity peaked 48 h after drug delivery.

### Tissue-distribution results of PTX in different groups

**Fig. 5:**
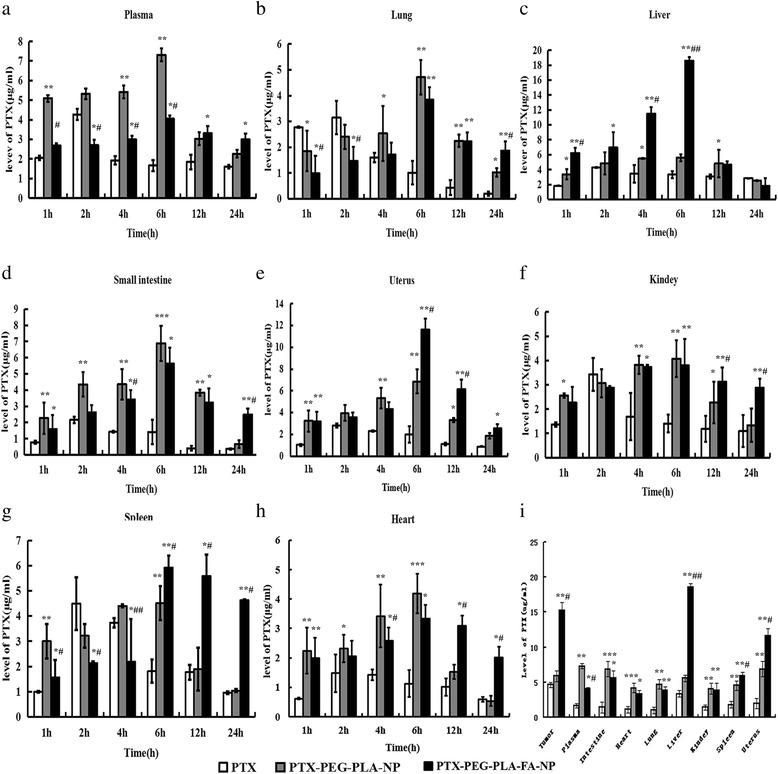
The tissues distributions of PTX in different groups in tumor bearing mice. Notes: The PTX levels in different tissues at various time points after three kinds of PTX solutions administration. (**a**) plasma; (**b**) lung tissues; (**c**) liver tissues; (**d**) small intestine tissues; (**e**) uterus tissues; (**f**) kidney tissues; (**g**) spleen tissues; (**h**) heart tissues; (**i**) PTX levels of different groups in different tissues at 6 h. The PTX concentrations at 6 h in different tissues were much higher in the nano-carriers groups than in the free PTX group. The PTX concentration tumor tissues and reticuloendothelial system in PTX-PEG-PLA-FA-NP group were higher than that of other two groups. *p < 0.05, **p < 0.01, ***p < 0.001 vs PTX. ^**#**^*p* < 0.05, ^**# #**^*p* < 0.01, ^**# # #**^*p* < 0.001 vs PTX-PEG-PLA-NP

**Fig. 6:**
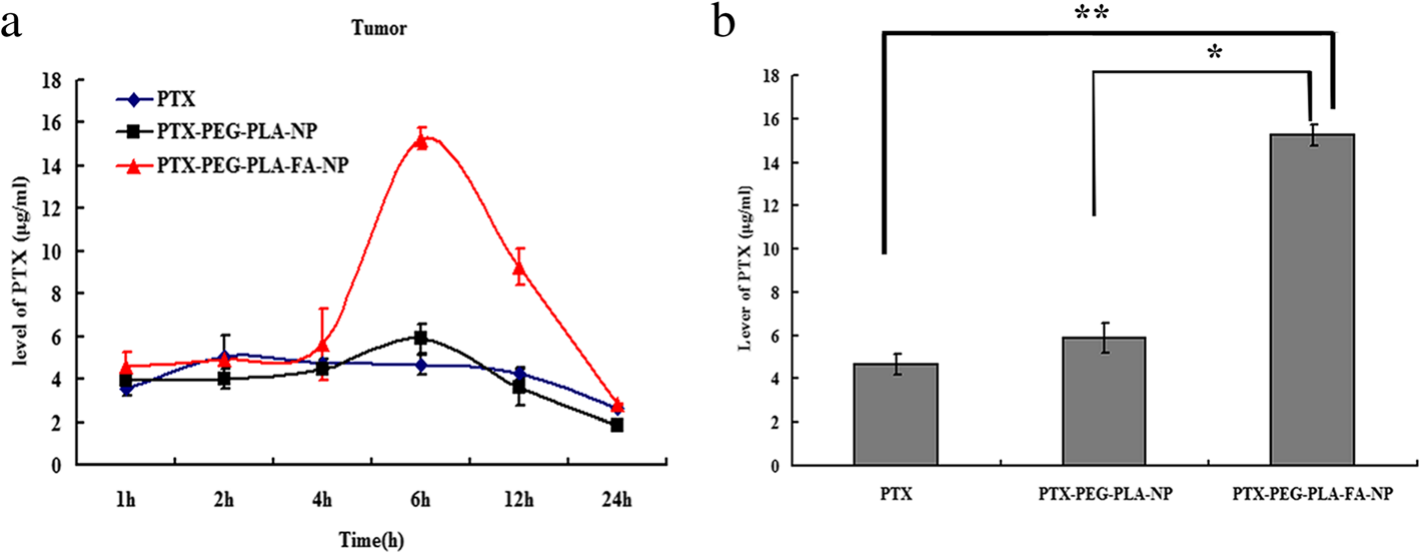
The tissue distributions of PTX in tumor tissues in different drug groups. Notes: The PTX levels in tumor tissues at various time points after drug delivery by three kinds of PTX solutions. (**a**) The PTX concentration of three groups in tumor tissues; (**b**) The PTX concentration in tumor tissues at 6 h. The PTX concentrations at various time points were much higher in the PTX-PEG-PLA-FA-NP group than free PTX and FA-free nano-carriers group, which suggested that the PTX-PEG-PLA-FA-NP could accumulate selectively in tumor tissues. For example, the PTX level in the PTX-PEG-PLA-FA-NP group at 6 h (15.267 ± 0.475 μg/ml) was much higher than that of PTX-PEG-PLA-NP group (5.883 ± 0.674 μg/ml) and PTX group (4.663 ± 0.357 μg/ml) (*p* < 0.05). *p < 0.05, **p < 0.01, ***p < 0.001

The PTX levels in plasma and other tissues at different time points after drug delivery are shown in Fig. 5. First, the peak reach times were extended in both nano-carrier groups (6 h) compared to the free PTX group (2 h) in all tissues, including the tumor tissue, as shown in Fig. 6a. These results once again confirmed the slow-release effect of nano-carriers similar to the pharmacokinetics study results. Second, in tumor tissues, the PTX concentrations in the PTX-PEG-PLA-FA-NP group (15.27 ± 0.48 μg/ml) at 6 h after drug delivery were much higher than in the free PTX (4.66 ± 0.36 μg/ml, *p* < 0.05) and PTX-PEG-PLA-NP groups (5.88 ± 0.67 μg/ml, *p* < 0.05), as shown in Fig. 6b, while there was no statistical difference between the latter two groups. We also obtained the same results at other time points. All these results indicated the tumor-targeting characteristic of PTX-PEG-PLA-FA-NP based on FA-mediated active targeting. Third, except for the liver, uterus, and tumor tissues, PTX-PEG-PLA-NP had greater uptake than in the PTX-PEG-PLA-FA-NP group by other tissues at most time points. Interestingly, the PTX concentration was at low levels in the kidney, heart, lungs, and small intestine in the PTX-PEG-PLA-FA-NP group compared to the liver and uterine tissues.

### Therapeutic anti-tumor effects of different groups in vivo

**Fig. 7:**
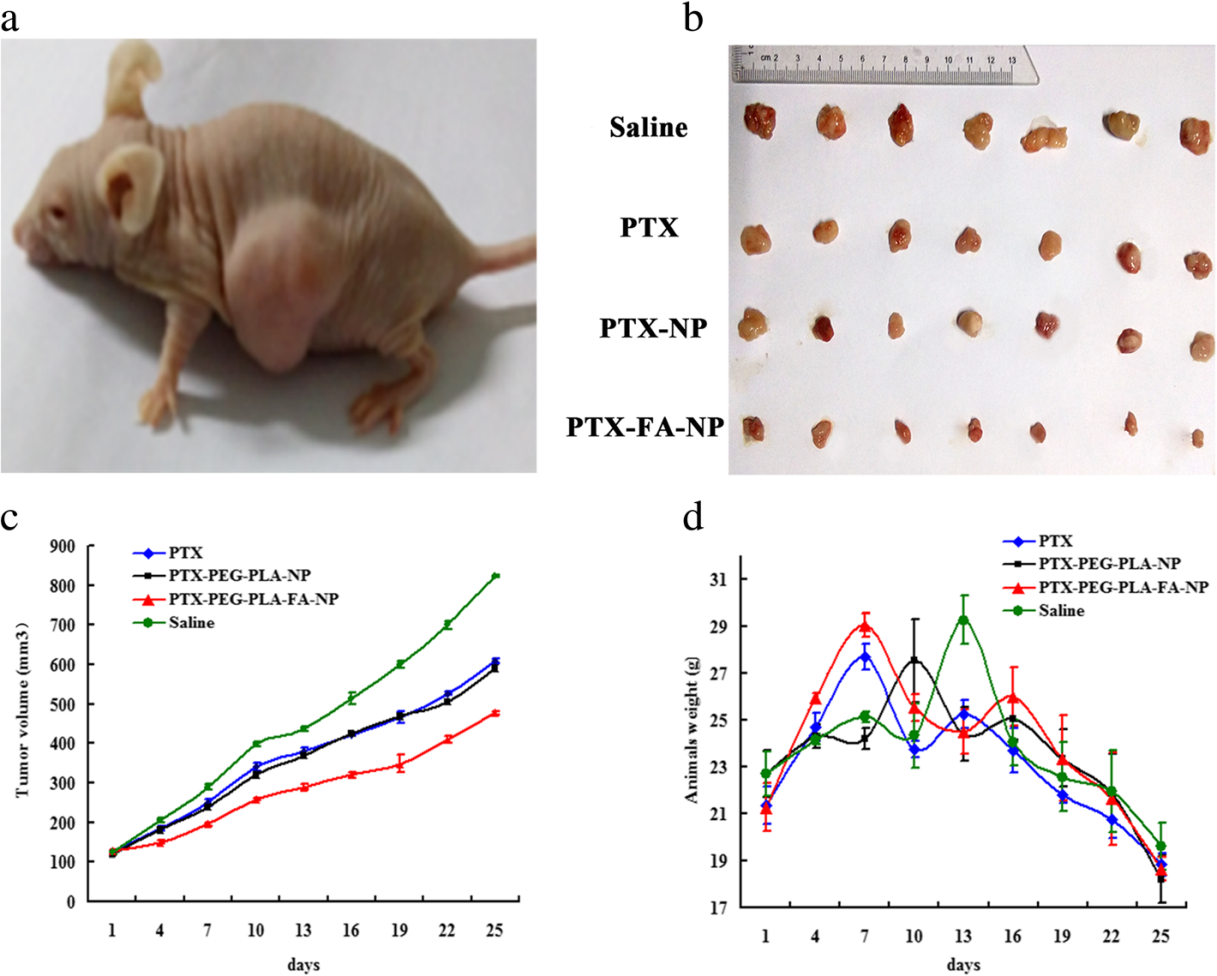
Anti-tumor effect of free PTX, PTX-PEG-PLA-NP and PTX-PEG-PLA-FA-NP solutions to the nude mice with subcutaneous tumor. Notes: (**a**) Established subcutaneous tumor models in nude mice; (**b**) Tumor tissues removed from the mice at the end point in different groups; (**c**) Tumor growth curves throughout the whole experiment. The inhibitory rates was 41.88% in the PTX-PEG-PLA-FA-NP group, which was 1.5 times higher than that in PTX-PEG-PLA-NP group (28.02%) and free PTX group (25.99%); (**d**) Body weight change of tumor-bearing mice. There was no significant difference in the average body weights among four groups (*p* > 0.05), which means no obvious side effects of nano-carriers system

The final tumor sizes in the treated mice were notably reduced in the PTX-PEG-PLA-FA-NP group, as seen in Fig. 7b, while there was no statistical difference between the free PTX and PTX-PEG-PLA-NP groups (*p* > 0.05). The final tumor size in the PTX-PEG-PLA-FA-NP group was 477.89 ± 4.66 mm^3^, which was much smaller than in the PTX-PEG-PLA-NP (591.89 ± 9.37 mm^3^, *p* < 0.05) and free PTX (608.38 ± 6.05 mm^3^, *p* < 0.05) groups as well as the control group (822.31 ± 43.10 mm^3^, *p* < 0.05). All treatment groups exhibited obvious anti-tumor effects compared to the control group (*p* < 0.05). The tumor growth curve showed that the PTX-PEG-PLA-FA-NP group had a much stronger anti-tumor effect than the other groups, as shown in Fig. 7c. The tumor inhibitory rates based on tumor volume were 41.88% in the PTX-PEG-PLA-FA-NP group, which was around 1.5 times higher than in the PTX-PEG-PLA-NP (28.02%) and free PTX groups (25.99%). Figure 7d shows the body weight changes of the animals. There was no significant difference among the four groups (*p* > 0.05), which indicated that there were no obvious side effects in the nano-carriers group.

## Discussion

In this study, we developed a novel active-targeting drug-delivery system, PTX-PEG-PLA-FA-NP, which is a potential effective and promising therapeutic strategy for cancer treatment. Polylactic acid (PLA) is a synthetic biodegradable polymer that has been approved by the U.S. Food and Drug Administration (FDA) for medical applications [18, 19]. However, because of its weak hydrophilicity, excessively long degradation time, and low drug loading of polar drugs [20, 21], PLA’s applications are limited. To overcome these shortcomings, PEG was combined with PLA to form a block co-polymer in this study [22–24]. Most kinds of cancer including ovarian cancer over-express FA receptor, and FA was demonstrated to be an excellent tumor-targeting molecule in nano-carrier drug delivery systems [25, 26]. In this study, we attached the FA molecule to the PEG-PLA block co-polymer by covalent bonding between the hydroxyl of the FA molecule and the N-terminal of PEG-PLA and eventually developed the PTX-loaded active tumor-targeting drug-delivery system PTX-PEG-PLA-FA-NP.

Paclitaxel is usually solubilized in polyoxyethylene castor oil (Cremophor EL) and ethanol (1: 1) for clinical application because of its poor hydrophilicity [27, 28], which frequently induces anaphylactic shock. In this study, PTX was loaded into the nano-carriers, and the problem of poor hydrophilicity and anaphylactic response was thus resolved. This was one of the advantages of our PEG-PLA-FA-NP drug-delivery system.

Another advantage of our nano-delivery system was its perfect slow-release effect*.* The drug release study, the pharmacokinetic study, and the drug-distribution study results demonstrated that PTX loaded into nano-carriers could be sustained for a much longer time (the prolonged *t*_*1/2*_ and peak reach time) in the body circulation than the free PTX solutions. In other words, given the same drug dose, nano-carriers could make PTX more effective against tumors.

**Fig. 8:**
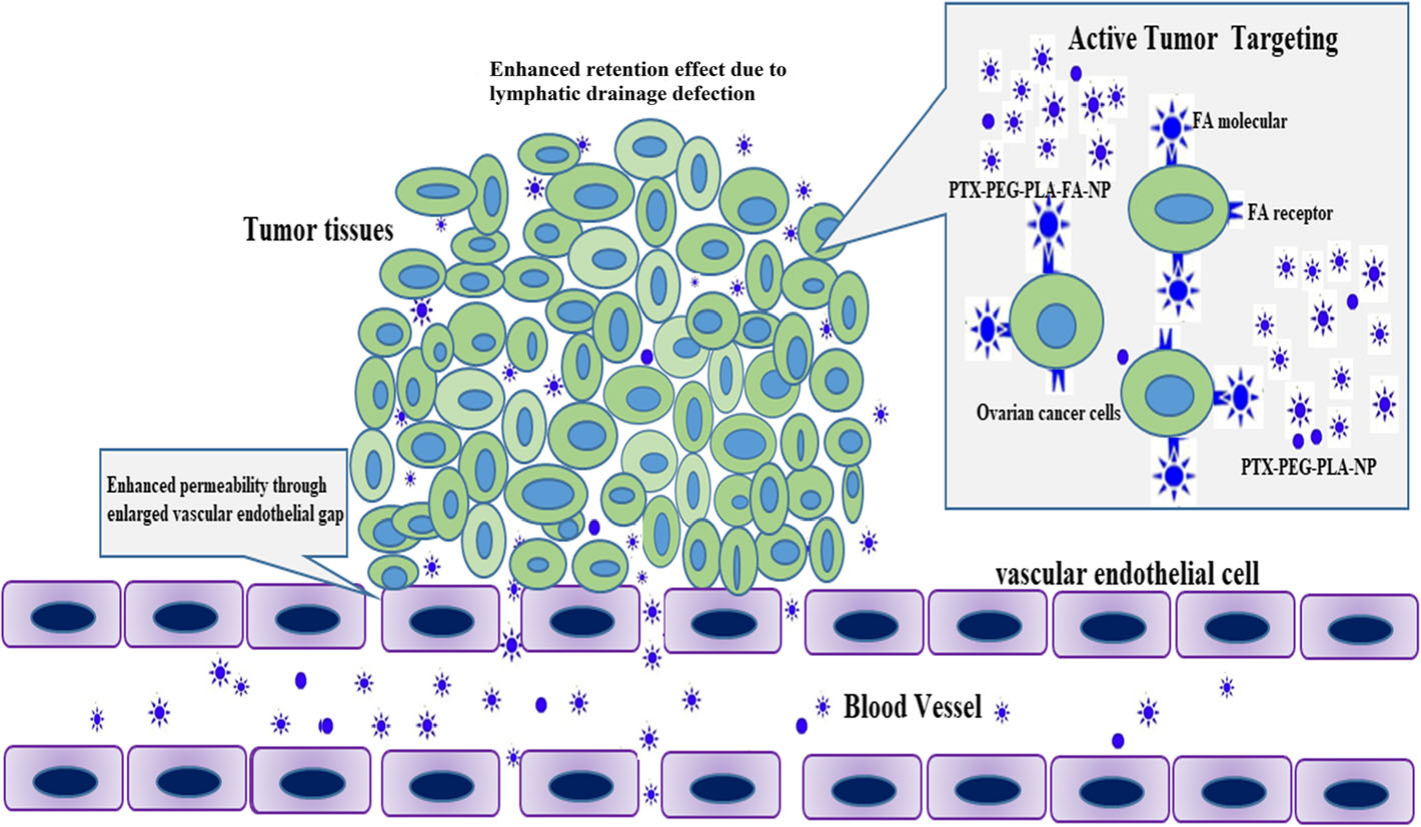
The schematic illustration of tumor targeting. Notes: This figure showed FA mediated active tumor-targeting mechanism and EPR effect

**Fig. 9:**
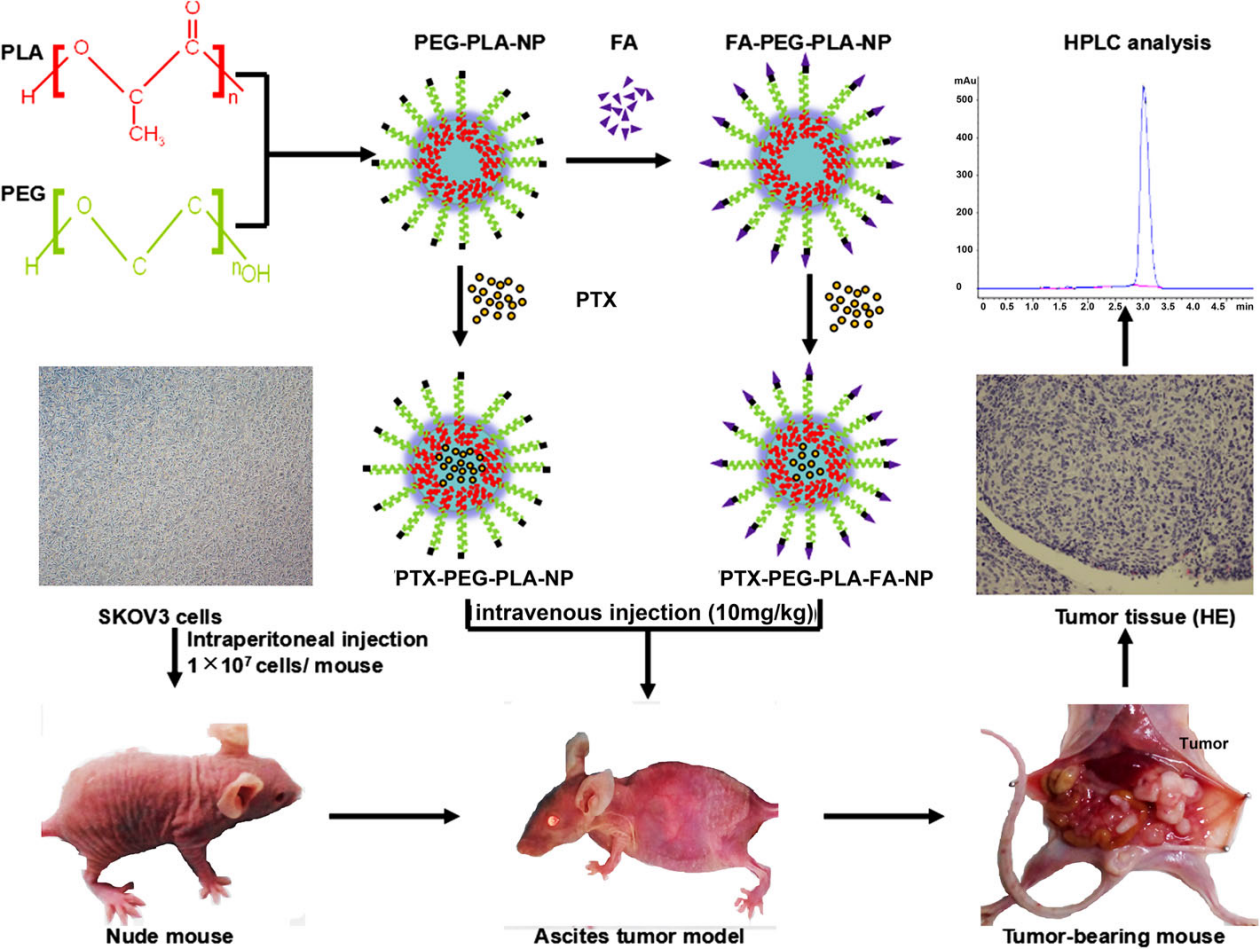
The flowchart of tissue-distribution study in vivo

The most important advantage of our nano-delivery system was its active targeting characteristic of tumor tissues. In contrast to FA-free PTX-PEG-PLA-NP, PTX-PEG-PLA-FA-NP had greater uptake by SK-OV-3 cells in vitro. A drug distribution study of tumor-bearing animals showed that the PTX concentration in tumor tissues in the PTX-PEG-PLA-FA-NP group was 3 times higher than in the FA-free PTX-PEG-PLA-NP and PTX groups. One possible reason is that the FA molecule can be recognized by the FA receptor on the surface of the cancer cells and induces receptor-mediated endocytosis, which is supposed to be the basic theory of the active tumor-targeting effect of PTX-PEG-PLA-FA-NP. Another reason for this difference might contribute to the enhanced permeability and retention (EPR) effect of nano-carriers in the body’s circulation [29]. A schematic illustration of tumor targeting was shown in Fig. 8. Figure 9 indicated that the flowchart of tissue-distribution study in vivo. There are also many other factors affecting the distribution of nano-carriers in vivo, such as the target organ type, the nano-carriers’ size, and the surface charges of the nano-carriers [30].

The excellent slow-release effects and tumor-targeting characteristics significantly enhanced the anti-tumor efficiency of PTX-PEG-PLA-FA-NP for ovarian cancer, which was also confirmed in this study. PTX-PEG-PLA-FA-NP was proved to be more toxic to SK-OV-3 cells in vitro at different dosages than PTX-PEG-PLA-NP and PTX. Compared to free PTX solutions, half-dose PTX in PTX-PEG-PLA-FA-NP could achieve the same toxicity to cells. In tumor-bearing animals, the tumor inhibitory rate was 41.88% in the PTX-PEG-PLA-FA-NP group, which was approximately 1.5 times higher than that in the PTX-PEG-PLA-NP (28.02%) and free PTX groups (25.99%). All these results confirmed that PTX-PEG-PLA-FA-NP could significantly enhance the anti-tumor effects of PTX, which should contribute to the selective accumulation of PTX in cancer cells and tissues using a PTX-loaded nano-delivery system. Zhang et al. used FSH33 as a targeting molecule and prepared PTX-loaded FSH33-PEG-PLA-NP, which showed tumor-targeting effects and nano-carrier-mediated toxicity only in vitro [31]. In our study, the pharmacokinetics analysis was done in Sprague-Dawley rats while the anti-tumor efficacy of the nano-carriers was determined using mice, there are two main reasons for using two different animal species. Firstly, There are two advantages that Sprague-Dawley rats as the animal model for pharmacokinetics analysis: 1. Nanoparticle solutions could be injected to Sprague-Dawley rats more easily through the tail vein than mice. 2. The pharmacokinetics analysis need the drug concentrations in blood at different time points. Sprague-Dawley rats have enough plasma to accomplish all time points experiment. So we choose Sprague-Dawley rats to do the pharmacokinetics study in vivo [32]. Secondly, in our study, we need tumor-bearing animal models to perform the drug distribution study and the anti-tumor experiment. Nude mice is immunodeficiency and thus suitable for tumor transplantation. So in this study, we choose nude mice to perform the drug distribution study and the anti-tumor effect experiment in vivo [33].

In our study, we confirmed the slow-release effect, tumor-targeting characteristics, and enhanced anti-tumor effects both in vitro and in vivo using an FA-enhanced PTX-PEG-PLA-NP drug-delivery system. Our study results offered more convincing evidence to support the application of nano-delivery systems against cancer. Conversely, we also found that more drugs could accumulate in the reticuloendothelial system (RES) organs. This indicated that we should pay more attention to the side effects of chemotherapy on RES organs when a nano-delivery system is used [34].

## Conclusion

In summary, this comprehensive study consisted of: (1) the preparation and characterization testing of nano-carriers, (2) the slow-release effect verification in vitro and in vivo, (3) the tumor-targeting characteristics verification in vitro and in vivo, and (4) the antitumor effect verification in vitro and in vivo. All these results showed that the PTX-PEG-PLA-FA-NP nano-delivery system had improved hydrophilicity, slow-release effects, low toxicity, and tumor-targeting characteristics. More drugs could be delivered into tumor tissues selectively and accordingly and the anti-tumor effect was enhanced significantly using this nano-delivery system. PTX-PEG-PLA-FA-NP-based therapy should be a promising new treatment strategy for ovarian cancer patients in the future.

## Additional files

Additional file 1: STR for A2780, SK-OV-3 and HO-8910 cells. (DOC 75 kb)

Additional file 2 DAPI fluorescence density of two nanocarriers in three different ovarian cancer cell lines. (PNG 971 kb)

## Abbreviations

AUC: Area under the curve
DCM: Dichloromethane
DLS: Dynamic light scatting
EO: Epoxy ethane
EPR: Enhanced permeation and retention
FA: Folic acid
FBS: Fetal bovine serum
HNMR: Nuclear magnetic resonance spectroscopy
HPLC: High performance liquid chromatography
KHMDS: potassium bis (trimethylsilyl) amide
M-mlV: moloney–murine leukemia virus
MTT: (3-[4,5-dimethylthiazol-2-yl]-2,5-diphenyltetrazolium bromide
NP: Nanoparticles
PBS: Phosphate-buffered saline
PEG-PLA: Polyethylene glycol and polylactic acid
PTX: Paclitaxel
RES: Reticuloendothelial system
TEM: Transmission electron microscopy
THF: Tetrahydrofuran
ZP: Zeta potential
