# Supplementary figures and images for: Development and evaluation of novel tumor-targeting paclitaxel-loaded nano-carriers for ovarian cancer treatment: in vitro and in vivo
Source: J Exp Clin Cancer Res. 2018 Feb 26;37:29. doi: 10.1186/s13046-018-0700-z (PMC6389131; doi:10.1186/s13046-018-0700-z)

**A2780-STR**


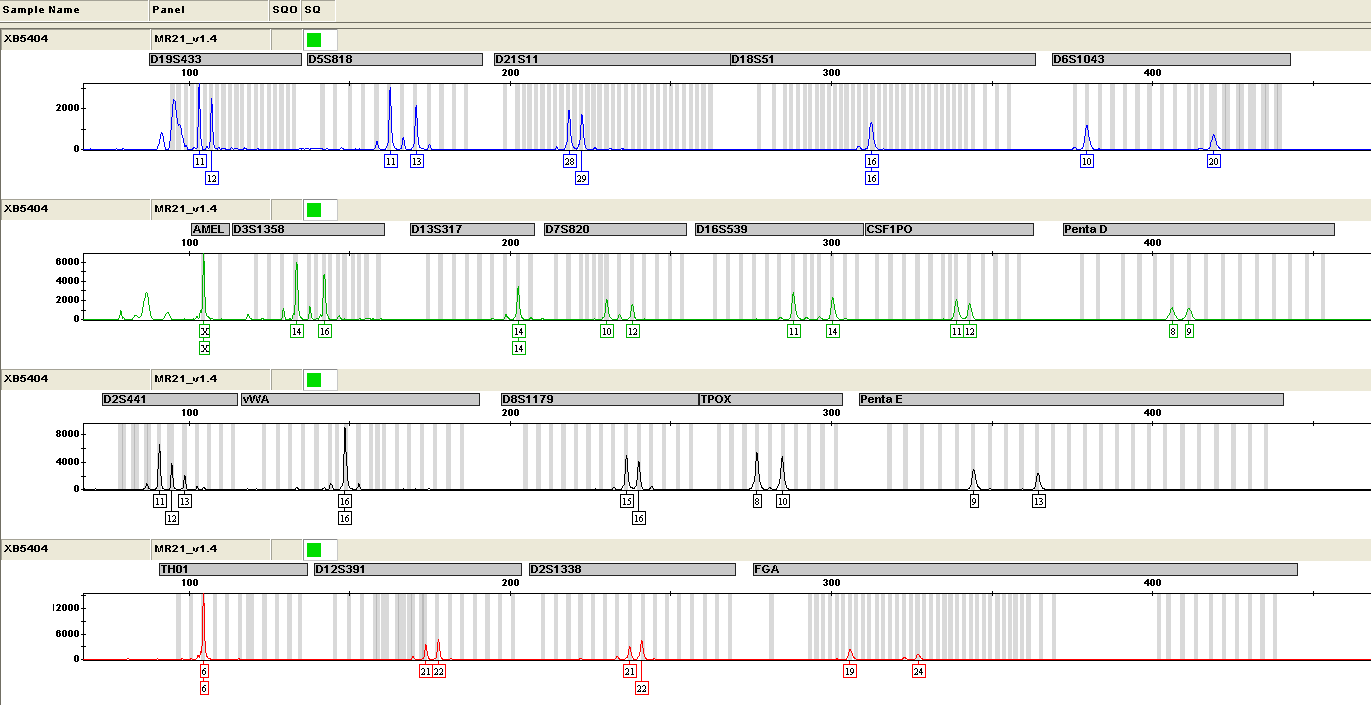


**SK-OV-3-STR**


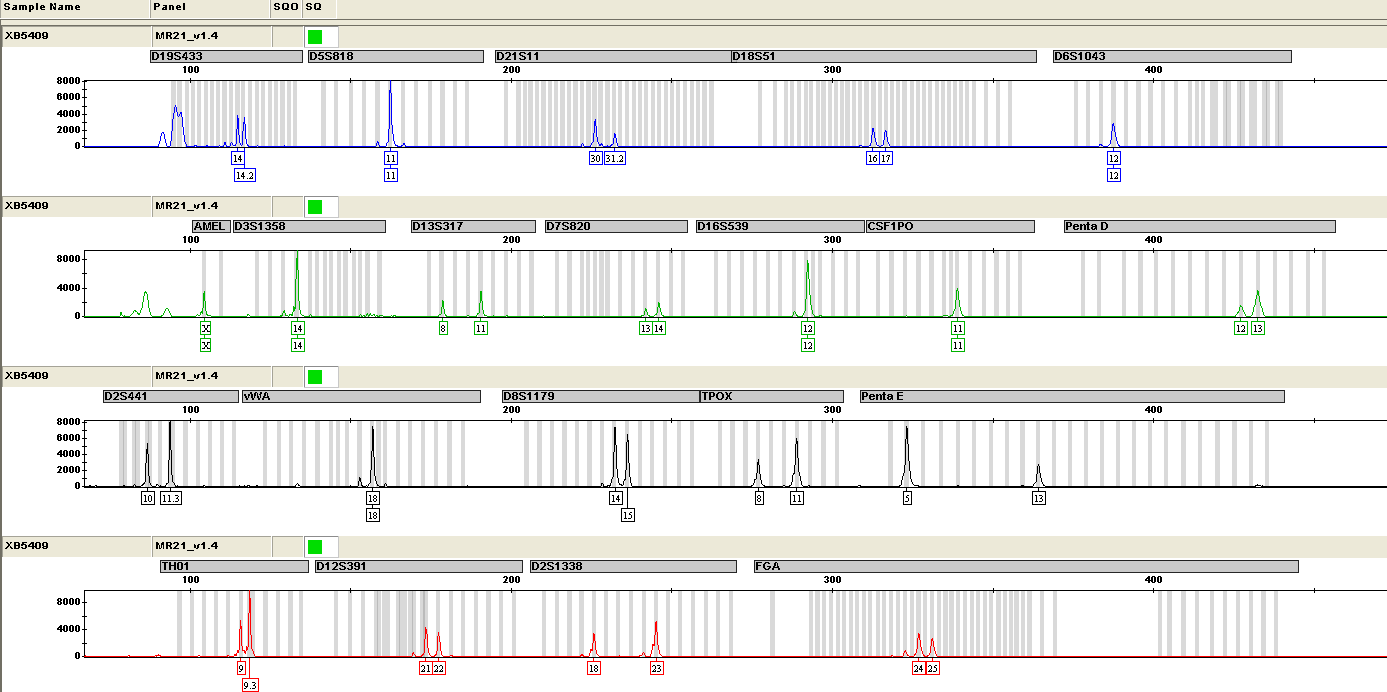


**HO-8910-STR**


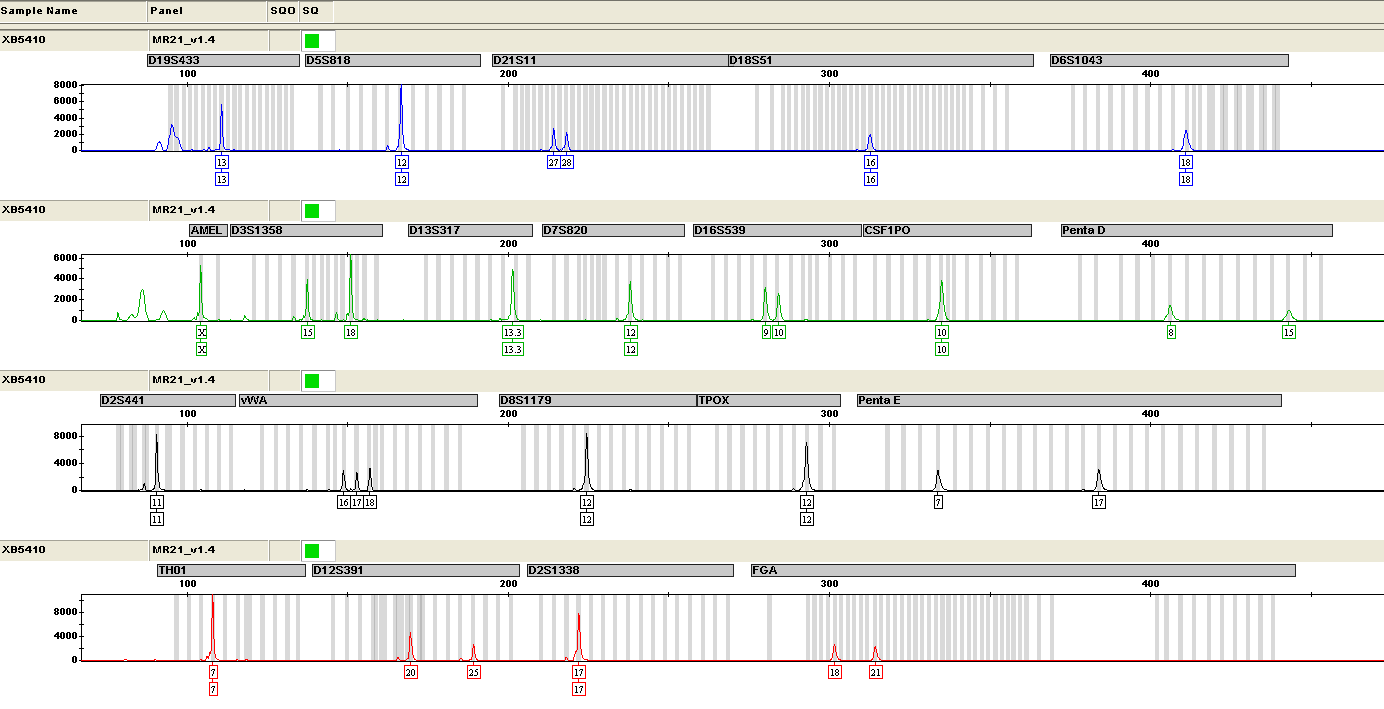

Supplement: Supplementary file 1 — STR for A2780, SK-OV-3 and HO-8910 cells. (DOC 75 kb) [file 13046_2018_700_MOESM1_ESM.doc]

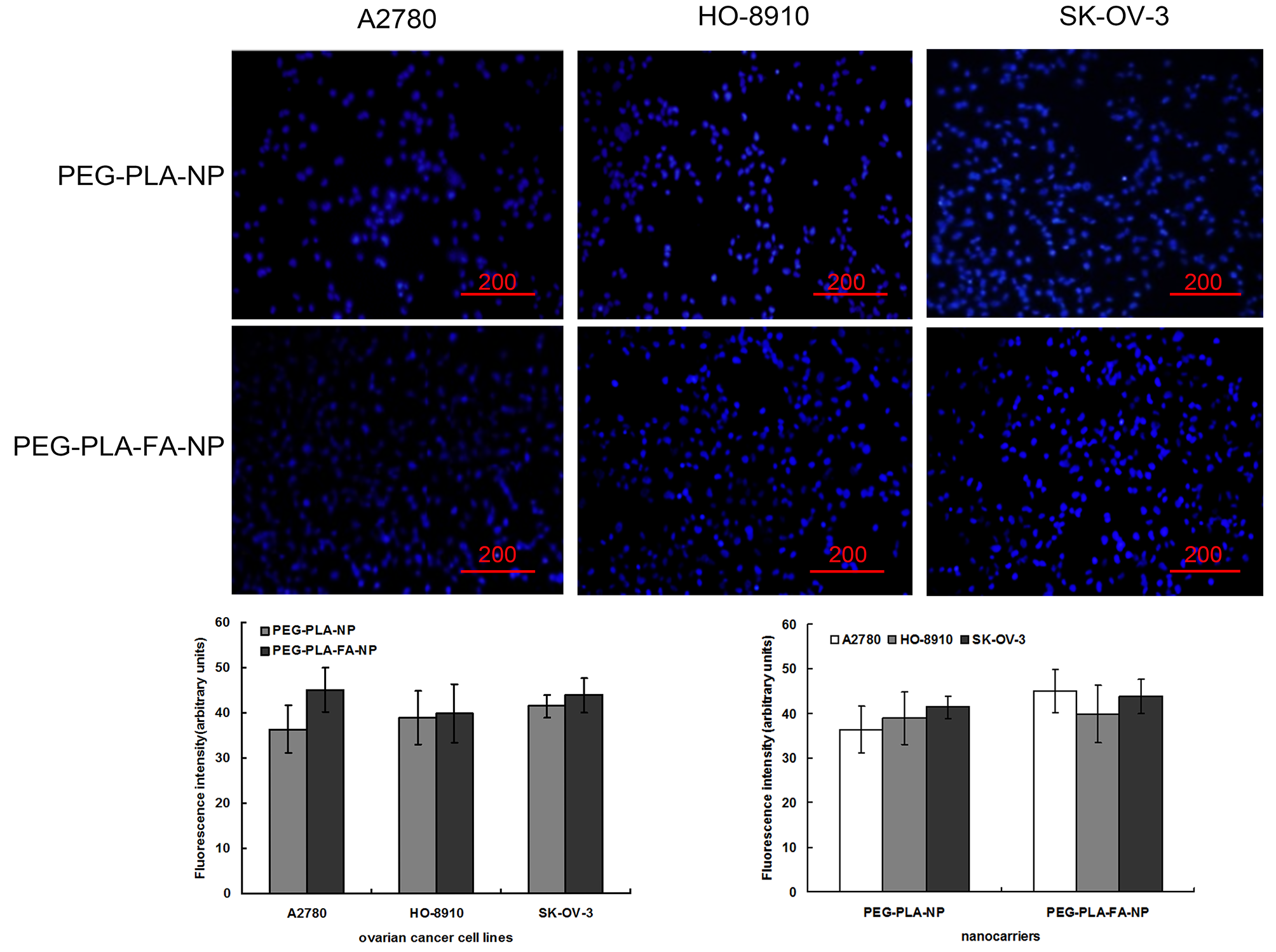

Supplement: Supplementary file 2 — DAPI fluorescence density of two nanocarriers in three different ovarian cancer cell lines. (PNG 971 kb) [file 13046_2018_700_MOESM2_ESM.png]
